# Variation in antiviral immunity and inflammation pathways precedes HIV-1 infection in a high-risk African cohort

**DOI:** 10.1172/JCI195172

**Published:** 2026-02-12

**Authors:** Mwikali Kioko, Shaban Mwangi, Lynn Fwambah, Amin S. Hassan, Jason T. Blackard, Philip Bejon, Eduard Sanders, Thumbi Ndung’u, Eunice W. Nduati, Abdirahman I. Abdi

**Affiliations:** 1 Bioscience Department, Kenya Medical Research Institute-Wellcome Trust Research Programme, Kilifi, Kenya.; 2 Institute for Human Development, Aga Khan University, Nairobi, Kenya.; 3 Division of Gastroenterology & Hepatology, University of Cincinnati College of Medicine, Cincinnati, Ohio, USA.; 4 Centre for Tropical Medicine and Global Health, Nuffield Department of Medicine, and; 5 Sir William Dunn School of Pathology, University of Oxford, Oxford, United Kingdom.; 6 The Aurum Institute, Johannesburg, South Africa.; 7 Africa Health Research Institute, Durban, South Africa.; 8 HIV Pathogenesis Programme, Doris Duke Medical Research Institute, University of KwaZulu-Natal, Durban, South Africa.; 9 Ragon Institute of Mass General Brigham, Massachusetts Institute of Technology and Harvard University, Cambridge, Massachusetts, USA.; 10 Division of Infection and Immunity, University College London, London, United Kingdom.; 11 Pwani University Biosciences Research Centre, Pwani University, Kilifi, Kenya.

**Keywords:** AIDS/HIV, Immunology, Infectious disease, Biomarkers, Th1 response, Th2 response

## Abstract

**BACKGROUND:**

Susceptibility to HIV-1 infection varies between individuals, but the biological determinants of acquisition risk remain poorly defined.

**METHODS:**

We conducted a case-control study nested within a high-risk cohort in Kenya. We compared the plasma extracellular RNA collected before HIV-1 acquisition with that from matched uninfected individuals acting as controls to identify immunological processes linked to infection risk.

**RESULTS:**

Individuals who later acquired HIV-1 exhibited upregulation of immune processes that facilitate viral infection, including T cell suppression and type II IFN and Th2 immune responses. In contrast, processes associated with antiviral defence and tissue repair, such as neutrophil and NK cell responses, type I IFN responses, wound healing, and angiogenesis, were downregulated.

**CONCLUSION:**

These findings highlight dampened antiviral immunity prior to exposure as a correlate of increased risk for subsequent HIV-1 acquisition.

**FUNDING:**

This work was supported by a Wellcome Trust Award (209289/Z/17/Z) and the Sub-Saharan African Network for TB/HIV Research Excellence (SANTHE) through the Developing Excellence in Leadership, Training and Science in Africa (DELTAS Africa) programme (Del-22-007), which is supported by the Science for Africa Foundation; Wellcome Trust; the UK Foreign, Commonwealth & Development Office; the European Union; and the Ragon Institute of Mass General, MIT, and Harvard. The Bill & Melinda Gates Foundation, Gilead Sciences Inc., and Aidsfonds provided additional support. The US President’s Emergency Plan for AIDS Relief (PEPFAR) supported the cohort study through the US Agency for International Development (USAID).

## Abstract 2

People at high risk for HIV-1 infection in an African cohort show differences in immune responses before infection, which may influence HIV-1 acquisition

## Introduction

Susceptibility to HIV-1 infection varies significantly across populations and individuals (1–3). For example, analysis from multiple studies showed that sub-Saharan Africa has a higher risk of HIV-1 transmission per sexual contact compared with higher-income regions (1). Although these differences may reflect low access to antiretroviral drugs in sub-Saharan Africa at the time, intrapopulation differences in susceptibility have been documented in a longitudinal study of high-risk Kenyan adults, in which only 7% were infected during follow-up, despite likely widespread exposure (4). This variability stems from a diverse range of factors, including behavioral differences, viral load, characteristics of circulating viruses (including HIV-1 subtype), and host-related factors such as genetic diversity and environmental exposures, including sexually transmitted infections (STIs) that can modulate basal immune status (2, 5, 6). However, the specific host biological factors and preexisting pathogens associated with HIV-1 acquisition are not fully known.

Identifying biological determinants of HIV-1 susceptibility is crucial for developing diagnostic biomarkers and interventions (7, 8). High-throughput omics techniques, including proteomics and transcriptomics, are increasingly used to understand host mechanisms that predispose to HIV-1 infection (4, 9). Transcriptomics is a sensitive method for detecting subtle differences in gene expression, providing insights into the host’s immune response and immunomodulatory effects of pathogens (10).

All cells secrete a diverse population of RNA collectively called extracellular RNAs (exRNAs) into biofluids such as plasma, saliva, and urine (11, 12). The majority of these exRNAs are secreted within membrane-bound vesicles called extracellular vesicles (EVs), which protect them in the harsh extracellular space (11–18). Additionally, the profiles of circulating exRNAs largely reflect the biological state of the secreting cells, which provides a more holistic view of systemic biological processes (19–21) and pathogen signals (22–26) relative to the cellular RNA obtained from peripheral immune cells. Therefore, analyzing plasma-derived exRNA from preinfection samples may provide valuable immune correlates of HIV-1 acquisition.

Here, we highlight transcriptional immune correlates of HIV-1 susceptibility by retrospectively analyzing plasma-derived exRNA collected before HIV-1 infection in a case-control study nested within a longitudinal cohort of HIV-negative high-risk individuals in coastal Kenya (27).

## Results

### Plasma exRNA highlights immunological pathways associated with HIV-1 acquisition risk.

**Figure 1:**
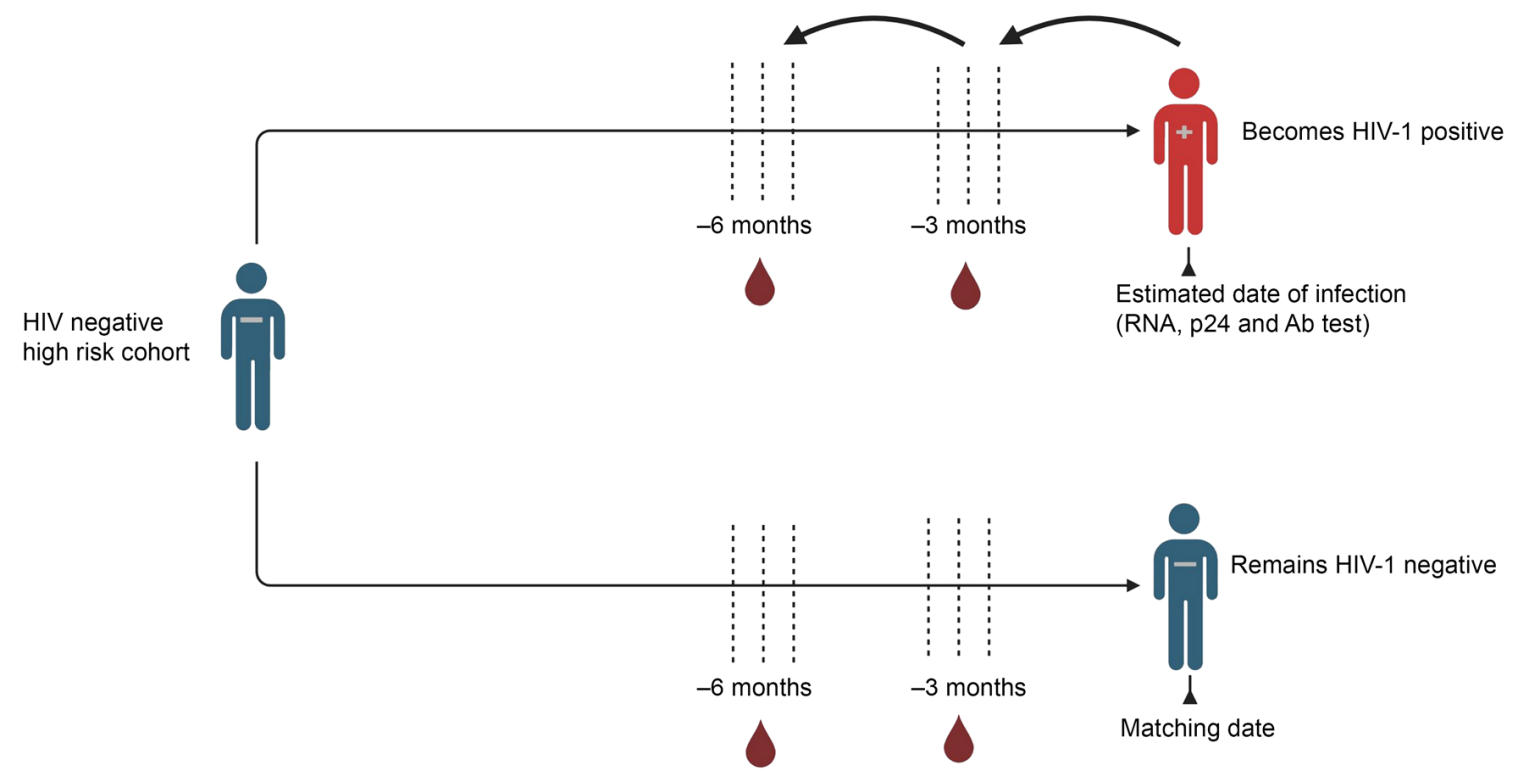
Schematic representation of our study design. Three and 6 months prior to HIV infection, samples were selected from a historic high-risk cohort study conducted on the Kenyan coast between 2006 and 2011. Cases were defined as those who tested positive for HIV during follow-up using RT-PCR, p24 antigen, and HIV-1–specific antibody assays. Controls were defined as individuals who remained HIV negative during follow-up; they were matched to the cases based on sex, age, risk group, follow-up duration, and availability of samples.

**Figure 2:**
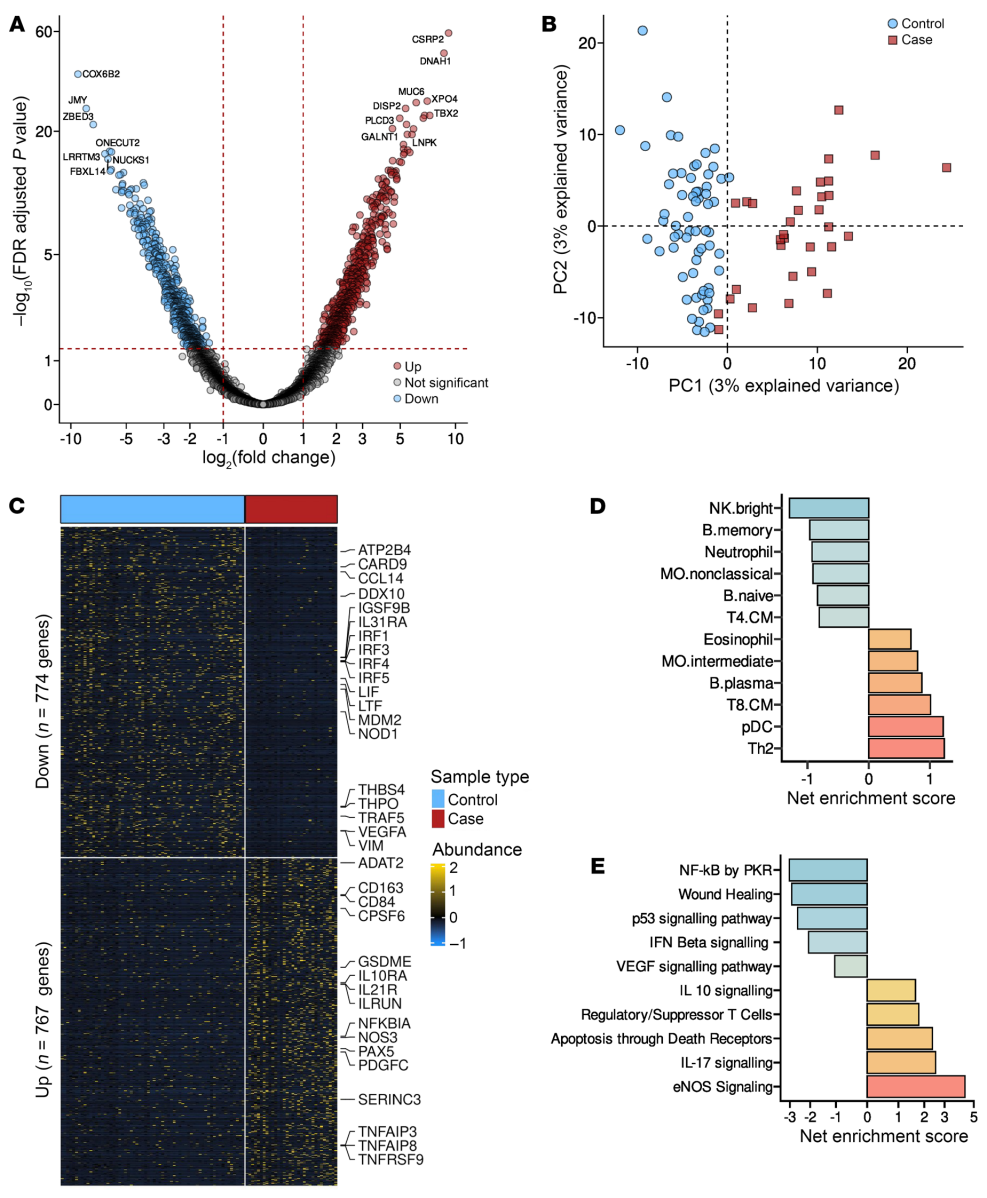
Cases exhibited a deregulated immunological profile 3 months prior to HIV-1 infection. (**A**) Volcano plot showing differentially altered genes between 32 cases and 64 controls, 3 months prior to cases being HIV positive. Red dots represent genes upregulated in cases, blue dots represent downregulated genes, and grey dots represent unaltered genes. (**B**) The differentially altered genes can distinguish HIV-1 cases from those who remained negative. (**C**) Supervised heatmap clustering showing differences in gene expression between cases and controls. (**D**) Gene enrichment analysis showing transcriptional alteration at the cellular level. Genes belonging to neutrophils and NK cells were downregulated, while those belonging to eosinophils and Th2 cells were upregulated. (**E**) Pathway gene enrichment analysis shows that immunosuppressive biological processes, such as IL10 signaling and Tregs, were upregulated in the cases, while inflammatory and reparative processes were downregulated.

The primary objective of this study was to identify preinfection transcriptional correlates of HIV-1 acquisition in high-risk adults. To achieve this, we took advantage of a long-term longitudinal cohort of high-risk individuals on the Kenyan coast, for whom the dates of HIV infection have previously been estimated (4, 27–29), as summarized in Figure 1 and described in detail in the Methods. We compared plasma-derived exRNA from individuals who later acquired HIV-1 (cases; *n =* 32), collected approximately 3 ± 2 months prior to the estimated date of infection (EDI), to that from matched negative individuals acting as controls (*n =* 64) (Figure 1). This analysis identified 767 genes with increased abundance and 774 genes with decreased abundance in HIV-1 cases, at a FDR of less than 5% (Figure 2A). Next, we performed principal component analysis and supervised heatmap clustering on the differentially enriched genes and found that the transcriptional profiles of EVs distinguished individuals acting as controls from the HIV-1 cases (Figure 2, B and C). The differentially increased genes included the endothelial nitric oxide synthase (*NOS3*), angiotensin-converting enzyme 2 (*ACE2*), IL-17 and IL-21 receptors (*IL17RA*, *IL17RD*, *IL21R*), the viral-sensing Toll-like receptor 7 (*TLR7*), and the inhibitor of *IRF3*- and NF-κB–dependent antiviral response gene (*ILRUN*) (30) (Figure 2C). In contrast, the differentially decreased genes featured the proangiogenic factor *VEGFA*, the IFN regulatory factors (*IRF1*, *IRF3*, *IRF4*, and *IRF5*), and the p53 negative regulator *MDM2* (Figure 2C).

Cell enrichment analysis demonstrated that the genes upregulated in HIV-1 cases 3 ± 2 months prior to infection belonged to cells such as eosinophils, plasma B cells, central memory CD8-T cells, plasmacytoid dendritic cells, and Th2 cells (Figure 2D). In contrast, the downregulated genes were enriched for signatures associated with several cell types, including NK cells, B memory cells, and neutrophils (Figure 2D). Next, we performed pathway enrichment analysis of the 767 genes increased in HIV-1 cases, revealing an overrepresentation of genes linked to NOS3, IL-17, and IL-10 signaling, suppressive T cell response, and apoptosis (Figure 2E). Conversely, the 774 genes that decreased in HIV-1 cases belonged to a wide range of biological pathways, including reparative processes (wound healing and p53 signaling) and pathways related to type I IFN, including NF-κB activation by protein kinase R (PKR) and IFN-β signaling (Figure 2E). These findings suggest that reduced type I IFN and proreparative immune responses, alongside elevated eNOS, suppressive T cell response, IL17 and IL10 signaling, are strongly linked to HIV-1 acquisition in high-risk adults.

### Plasma exRNA clustering uncovers distinct immunological endotypes in HIV-1 cases and individuals acting as controls.

**Figure 3:**
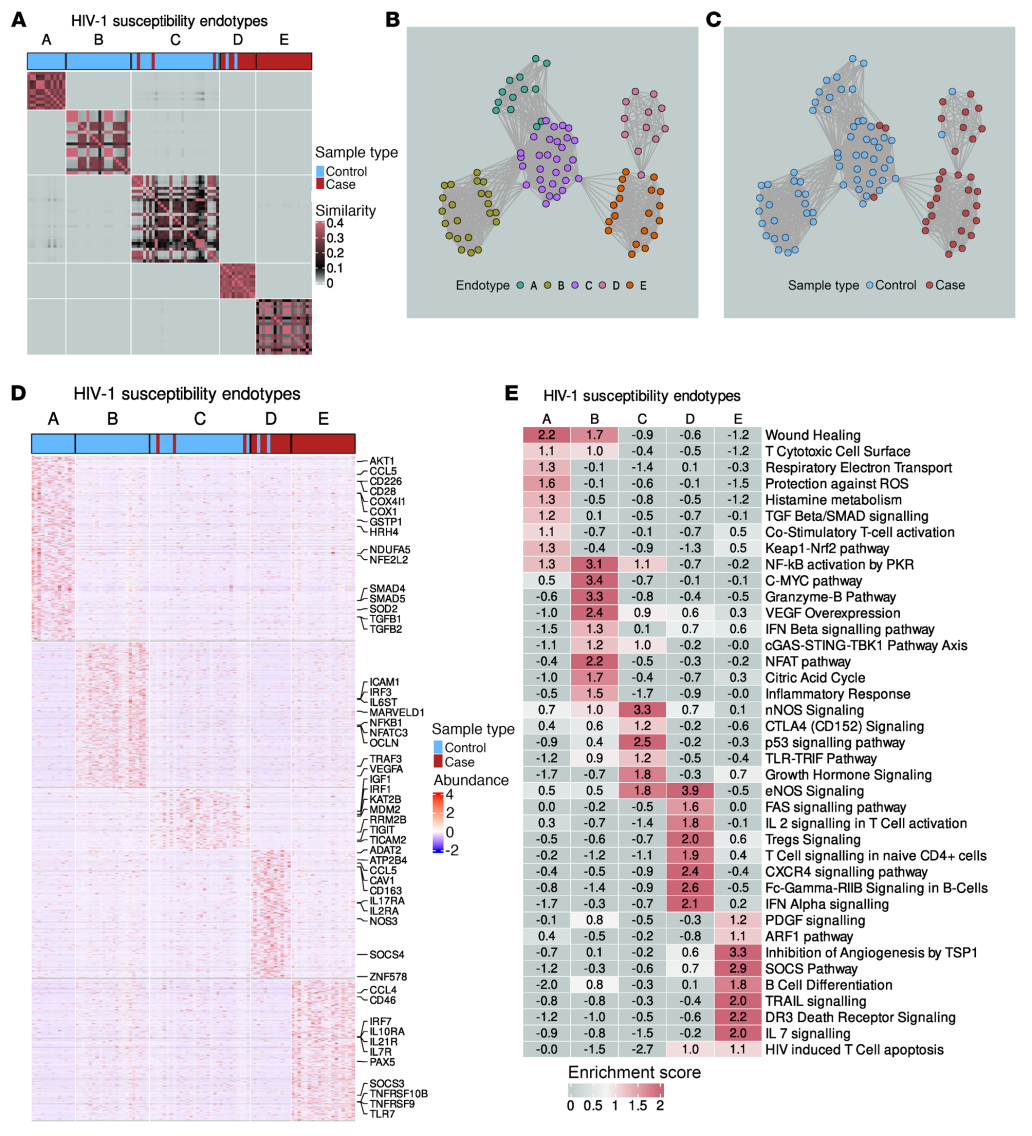
Cases and controls cluster into distinct immunological endotypes 3 months prior to HIV-1 infection. (**A**) Patient similarity matrix showing that EV RNA-seq data, 3 months prior to HIV-1 infection, splits controls and cases into 3 and 2 endotypes, respectively. (**B** and **C**) Patient similarity network colored by (**B**) endotype and (**C**) sample type. Each node represents a study participant, and each edge links 2 similar samples. (**D**) Heatmap clustering shows that the identified endotypes have distinct transcriptional profiles. (**E**) Heatmap showing the top pathways enriched in each endotype.

There may be heterogeneity in the biological mechanisms underlying protection or susceptibility to HIV-1 infection, which is obscured when comparing average biological signals between cases and controls. To reveal intragroup heterogeneity and biological signal, we constructed a participant similarity network using the exRNA dataset generated from the samples collected 3 ± 2 months prior to HIV-1 infection. Spectral clustering of the similarity network identified 5 endotypes of study participants — named A, B, C, D, and E — of which endotypes A, B, and C were enriched for individuals acting as controls, while D and E were enriched for HIV-1 cases (Figure 3, A–C). We subsequently performed differential feature analysis and identified over 4,000 genes whose exRNA profiles differed significantly between the endotypes, surpassing the differential signal observed in the case-control analysis (Figure 3D and Supplemental Table 1; supplemental material available online with this article; https://doi.org/10.1172/JCI195172DS1). Pathway enrichment analysis revealed that the control endotypes were enriched for features associated with proreparative processes (wound healing, TGF-β/SMAD signaling, VEGF overexpression, and histamine metabolism), T cell function (T cell CD3, T cytotoxic cell surface, costimulatory T cell activation, granzyme B pathway, and CTLA4 signaling), mitochondrial function (protection against ROS, Keap1-Nrf2, respiratory electron transport, and citric acid cycle), and type I IFN signaling (IFN β signaling pathway, cGAS-STING-TBK1 pathway, TLR-TRIF pathway, and NF-κB activation by PKR) (Figure 3E).

The 2 endotypes, composed mainly of HIV-1 cases (Figure 3, A–C), were also enriched for distinct pathways, with genes augmented in endotype D linked to eNOS signaling, Tregs, CXCR4 signaling, and FAS-mediated apoptosis (Figure 3E). Finally, endotype E showed evidence of increased apoptosis, including HIV-1–mediated T cell apoptosis and TRAIL and DR3 death receptor signaling. Signatures of B cell differentiation, IL-7 signaling, and suppressor of cytokine signaling (SOCS) were also enriched in endotype E (Figure 3E). Our endotyping analysis revealed more differentially expressed genes than the case-control analysis, suggesting that distinct biological mechanisms may promote or impede HIV-1 infection.

### The immunological processes observed at 3 ± 2 months were conserved at 6 ± 2 months prior to HIV-1 infection.

**Figure 4:**
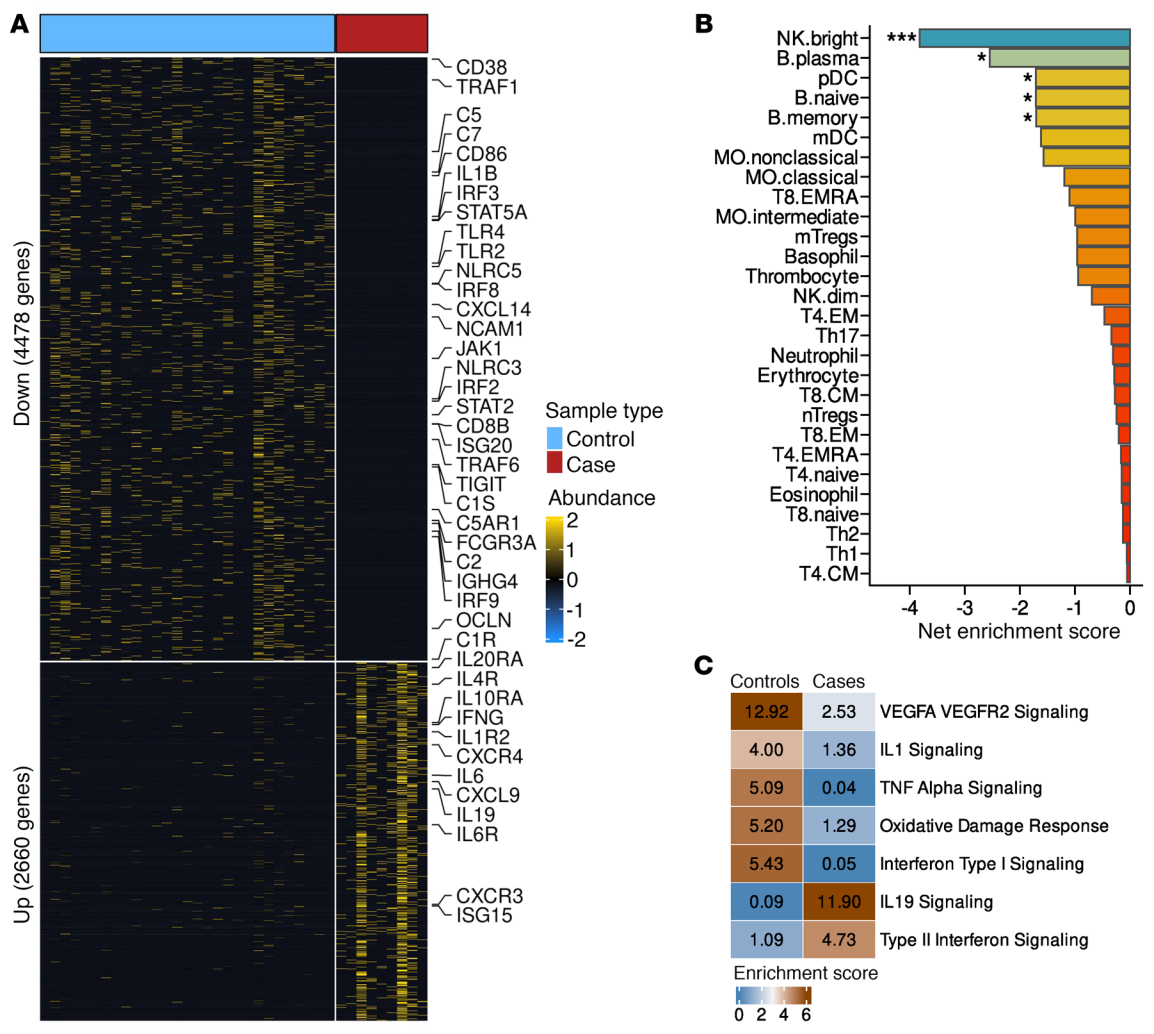
The immunosuppressive transcriptional profile is also evident 6 months prior to HIV-1 infection. (**A**) Heatmap showing differential gene expression between 9 cases and 29 controls 6 months prior to HIV-1 infection. (**B**) Genes belonging to NK cells and plasma B cell subsets were severely downregulated in HIV-1 cases relative to controls 6 months prior to infection *P = 0.05, ***P = 0.001. (**C**) Type II IFN response was upregulated in HIV-1 cases 6 months prior to infection, while type I IFN response pathways were upregulated.

To investigate whether the immune profile observed 1–5 months prior to HIV-1 infection was also evident at earlier time points, we analyzed the transcriptional profiles from 9 individuals who later acquired HIV-1 and 29 matched individuals acting as controls who remained uninfected, using samples collected 4–8 months before the cases became HIV-1 positive. We found that 2,688 genes were significantly increased in HIV-1 cases, while 4,521 genes were significantly decreased (Figure 4A and Supplemental Table 2). Cellular enrichment analysis of the altered genes showed significant downregulation of genes associated with NK cells (e.g., NCAM1, FCGR3A), plasma B cells (e.g., *CD38*), and plasmacytoid dendritic cells (Figure 4, A and B). When we performed pathway overrepresentation analysis, we observed that genes upregulated 6 ± 2 months prior to HIV-1 infection were enriched for type II IFN signaling (e.g., *CXCR3*, *IFNG*, *IL19*, and *CXCL9*) (Figure 4C). On the other hand, genes downregulated 6 ± 2 months prior to HIV-1 infection were enriched for type I IFN signaling (e.g. *IRF3, IRF9, JAK1, STAT2, STAT5A, IL1B, TLR2, TLR4*) and VEGFA-VEGFR2 signaling, consistent with the 3 ± 2 months prior to infection time point (Figure 4C). These observations confirm that reduced type 1 IFN–driven innate immunity, together with an elevated type II IFN state, precedes HIV-1 infection.

### The presence of human pegivirus type-1 is associated with HIV-1 acquisition.

**Figure 5:**
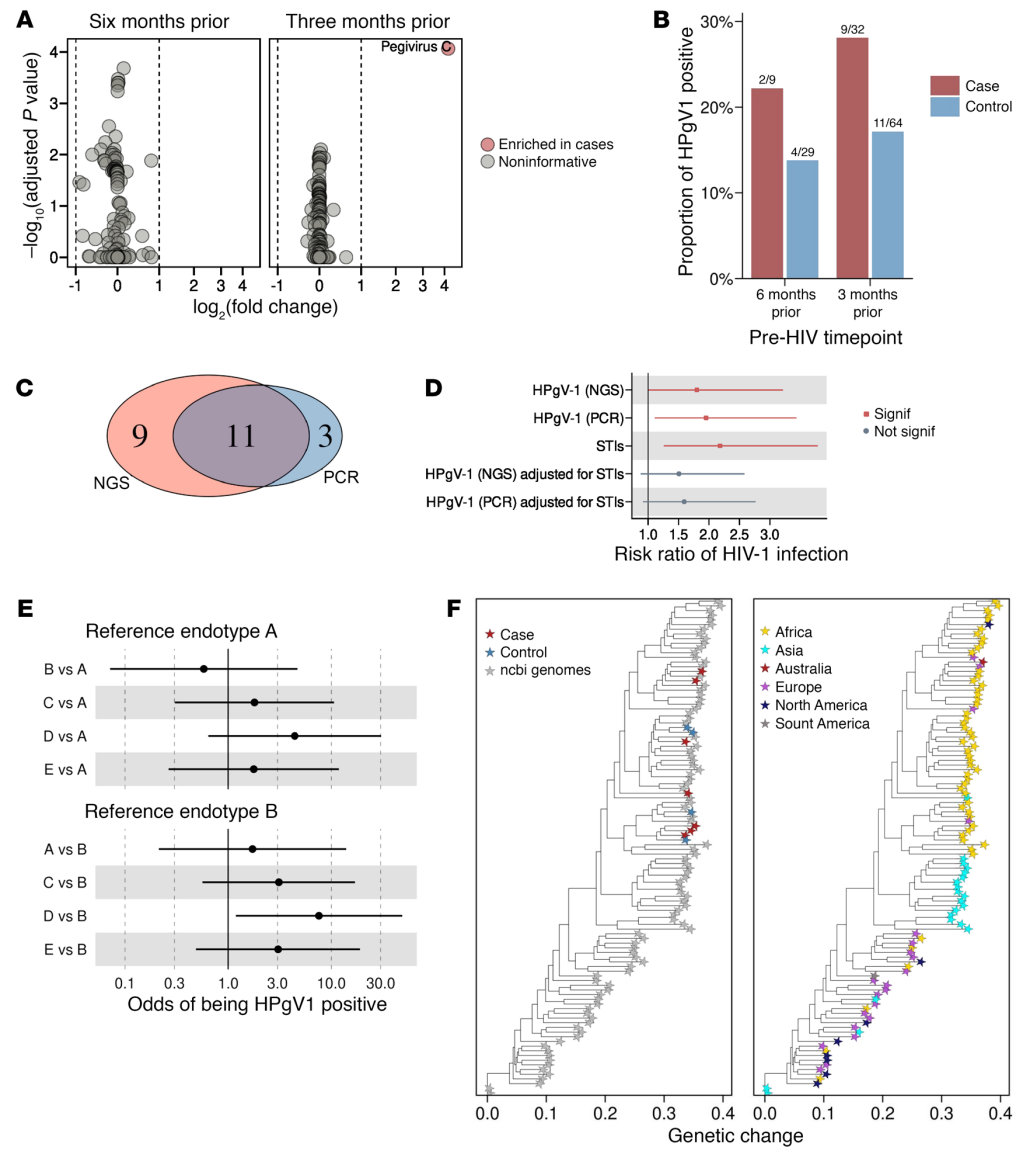
HPgV-1 infection predicts HIV-1 acquisition. (**A**) HPgV-1 RNA is more abundant in cases compared with controls at 3 months, but not at 6 months prior to HIV-1 infection. (**B**) Bar plots showing the proportion of HPgV-1–positive cases and controls. (**C**) Venn diagram showing the overlap between HPgV-1 detection by next-generation sequencing (NGS) and conventional PCR. (**D**) The presence of HPgV-1 3 months prior to infection is a nonindependent predictor of HIV-1 infection. (**E**) Forest plots comparing HPgV-1 status between the endotypes described in Figure 2. (**F**) HPgV-1 genomes exhibit regional clustering.

We next analyzed the exRNA-seq data using a metatranscriptomic approach to nominate potential pathogens associated with HIV-1 susceptibility. Human pegivirus type-1 (HPgV-1) RNA abundance was significantly higher in HIV-1 cases than in individuals acting as controls 3 months before HIV infection (log_2_ fold change > 4, FDR < 0.05) but not at 6 months (Figure 5A). Applying more stringent criteria (>5 reads) to define HPgV-1 positivity, rather than considering any detectable HPgV-1 RNA level as positive, we identified 20 HPgV-1 positive samples. HPgV-1 positivity was nonsignificantly higher among HIV-1 cases than among individuals acting as controls at both 3 months (28% in cases versus 17% in controls; OR = 1.89; 95% CI, 0.69–5.16; *P =* 0.29) and at 6 months (22% in cases versus 14% in controls; OR = 1.79; 95% CI, 0.27–11.86, *P =* 0.61), indicating a modest enrichment of HPgV-1 among individuals who later acquired HIV-1 (Figure 5B). 14 participants were identified as HPgV-1 positive by conventional PCR, of which only 3 of them were not detected using next-generation sequencing (NGS) (Figure 5C). Poisson regression analyses showed that HPgV-1 infection detected by NGS and PCR at 3 ± 2 months prior to HIV-1 infection was significantly associated with HIV-1 acquisition (NGS: relative risk [RR] = 1.99; 95% CI, 1.11–3.55; PCR: RR = 2.32; 95% CI, 1.32–4.08) (Figure 5D). However, after adjustment for other STIs, the association was reduced (NGS: RR = 1.51; 95% CI, 0.88–2.61; PCR: RR = 1.66; 95% CI, 0.96–2.87), indicating that HPgV-1 was not an independent predictor of HIV-1 acquisition. We next compared endotypes by HPgV-1 status, revealing that individuals in endotype D were more likely to be HPgV-1 positive than those in endotypes A or B (Figure 5E). To assess the effect of HPgV-1 on transcriptional differences between HIV-1 cases and individuals acting as controls, we compared transcriptional differences before and after adjusting for HPgV-1 status. We found a high correlation (R = 0.97, *P <* 0.0001) of the log_2_ fold changes before and after adjusting for HPgV-1 (Supplemental Figure 1 and Supplemental Table 3). Furthermore, 120 and 201 of the upregulated and downregulated genes between HIV-1 cases and individuals acting as controls, respectively, showed significant differential abundance between HPgV-1–positive and –negative individuals (Supplemental Figure 2 and Supplemental Table 4). We also compared transcriptional changes between HPgV-1–positive and –negative individuals within both the HIV case and the control groups and found overlaps of 37 (3.7%) and 38 (4.8%) upregulated and downregulated genes, respectively (Supplemental Figure 3 and Supplemental Table 5). Additionally, we reanalyzed previously published transcriptional data from PBMCs that were either exposed or unexposed to HPgV-1 in vitro (GSE131504) (31). The reanalysis revealed only 12 genes (6 upregulated and 6 downregulated) with concordant expression between exRNA and PBMCs (Supplemental Figure 4 and Supplemental Table 6).

Finally, we assessed the genetic relatedness of the HPgV-1 genome sequences from the samples at 3 ± 2 months prior to infection relative to samples from other parts of the world We generated 11 partial HPgV-1 genomes, of which 4 were from individuals acting as controls and 7 were from HIV-1 cases. We next performed phylogenetic analysis and found that the HPgV-1 genomes clustered by geographic origin, with our partial genomes coclustering with those from other African countries, consistent with the findings of previous studies (32) (Figure 5F).

## Discussion

In this study, we leveraged plasma-derived exRNA to determine preinfection immune correlates of HIV-1 acquisition among high-risk adults in a longitudinal cohort study (27, 29). We highlight key findings, explore their biological relevance to HIV-1 susceptibility, and offer potential avenues for future research and intervention. Given that the profiles of circulating exRNA often mirror molecular activities in the tissues most affected by a specific condition (19), in this case, the mucosal sites that serve as primary portals of HIV-1 entry, we also discuss our observations within the context of mucosal immune regulation.

Our differential feature analysis showed that, 3 months prior to HIV-1 infection, individuals who later got infected exhibited significant alterations in exRNA profiles compared with the individuals acting as controls. Notably, transcripts associated with IL-17 receptor signaling, apoptosis, Tregs, and eNOS signaling were upregulated in HIV-1 cases. Higher sexual activity, particularly receptive anal intercourse (33–35), together with STIs, may promote mucosal damage, immune activation, and apoptosis, events that compromise barrier integrity and facilitate viral entry. The elevated IL-17 receptor and eNOS signaling, along with Treg responses, may represent compensatory mechanisms that restore mucosal homeostasis (36–43) but could also be induced by STIs and anal intercourse (33, 44). However, chronic activation of these pathways could sustain inflammation and tissue damage. Moreover, enhanced IL-17 receptor signaling may also drive chemokines that enhance recruitment of key HIV-1 target cells — Th17 cells — at the mucosal sites (45–48). While Tregs help reduce immune activation, they are also susceptible to HIV-1 infection (49, 50) and can weaken antiviral response, collectively enhancing susceptibility to HIV-1 acquisition.

A key observation from our study was the downregulation of genes linked to type I IFN response, accompanied by an upregulation of type II IFN–associated transcripts in individuals who later acquired HIV-1. This pattern suggests a reprogramming of the immune landscape toward a less antiviral (30, 51–57) and more inflammatory state, which may increase the expression of key HIV-1 entry receptors such as CCR5 (58–60), thereby increasing susceptibility to HIV-1 infection. The suppression of type I IFN response may be driven by elevated IL-17 signaling, given that type I IFN and Th17 responses are known to act antagonistically (61). Indeed, individuals with a gain-of-function mutation in type 1 IFN signaling are predisposed to fungal infection due to impaired Th17 responses (62, 63), while chronic hyperactivation of Th17 responses has been associated with increased susceptibility to viral infections (45, 64, 65).

Our endotyping analysis identified 5 distinct endotypes, reflecting significant heterogeneity in the biological mechanisms at play. Three endotypes — A, B, and C — predominantly comprising samples from the control group, displayed immune profiles consistent with effective antiviral immunity (66, 67) and restrained immune activation, characterized by enhanced type I IFN response, T cell function, TGF-β/SMAD signaling, and oxidative phosphorylation. These features likely contribute to efficient antiviral defence (58) and maintenance of mucosal health. For example, increased TGF-β could confer protection against HIV-1 infection through maintaining an effective mucosal immune system impervious to viral entry (68, 69) or inhibiting the pro-HIV type II IFN immune response (70). In contrast, the two susceptibility endotypes, D and E, were enriched for Tregs and FAS-mediated signaling, TRAIL, and SOC3 pathways, signatures that suppress antiviral immunity and enhance mucosal disruption (71–73). Together, these findings suggest that preinfection immune heterogeneity, particularly involving the balance of IFN signaling and the interplay between T cell function, critically shapes HIV-1 acquisition risk and may inform precision prevention strategies. However, a larger study is necessary to identify the true heterogeneity of HIV-1 risk.

Our metatranscriptomic analysis identified HPgV-1 (also known as human pegivirus C type 1, GB virus C [GBV-C], or Pegivirus hominis) as significantly associated with HIV-1 acquisition, albeit, this association was less pronounced after adjusting for other STIs. HPgV-1 is a flavivirus that infects lymphocytes and NK cells and is transmitted by blood transfusion, sexual exposure, and mother-to-fetal transmission (74). While our data suggest that HPgV-1 is a correlate of HIV-1 acquisition, its predictive value is influenced by the presence of other STIs. This suggests that HPgV-1 may not directly drive susceptibility but instead reflects a permissive host immune environment conducive to sexually transmitted viral infection, thus representing a biomarker for HIV-1 risk.

Interestingly, during established HIV-1 infection, HPgV-1 has been linked to slower progression to acquired immunodeficiency syndrome (AIDS) (75–79). A plausible explanation, consistent with our data, is that HPgV-1 exploits an immune milieu characterized by reduced type I and elevated type II IFN responses (80), an immune balance that favors viral acquisition but limits immunopathology (81–84). However, a direct role for HPgV-1 in modulating host immunity cannot be ruled out, as suggested by other studies (26, 85–87).

The retrospective design of our study represents a key limitation. Concurrent collection of mucosal samples alongside blood would have allowed direct validation of the immunological signatures inferred from exRNA analyses against local mucosal responses. Consequently, some of our interpretations, although supported by existing literature, remain speculative and require confirmation through prospective studies.

In summary, we highlight the strength of plasma exRNA-seq in uncovering preinfection biological correlates of HIV-1 acquisition. Future research should focus on validating HPgV-1’s predictive value in larger cohorts. In conclusion, understanding the biological drivers of HIV-1 susceptibility among high-risk populations could enhance the development of prevention and treatment strategies.

## Methods

### Sex as a biological variable

Samples from cases and controls were obtained from both men and women. In our study, sex was not considered a biological variable of interest.

### Study design and population

#### Samples collected 3 ± 2 months prior to HIV infection.

A case-control study nested in a historic HIV-1 high-risk cohort from coastal Kenya was conducted. HIV-1–negative high-risk volunteers, including men who have sex with men and female sex workers aged ≥18 years, were recruited and followed from 2006 to 2011 for HIV-1 vaccine preparedness studies. Volunteers were screened for incident HIV-1 infection during follow-up using RT-PCR, p24 antigen, and HIV-1–specific antibody assays as previously described (4, 28). For any volunteer testing HIV-1 positive, an EDI was calculated to be 10 days before a positive HIV-1 RNA test (if antibody negative), 14 days before a p24 antigen–positive test (if RNA test was missing), or midway between the last negative and first positive HIV-1–specific antibody test (if both RNA and p24 tests were missing). Cases were defined as volunteers who tested HIV-1 positive, while controls were defined as individuals who remained negative at the end of a similar follow-up period (4). Plasma samples from cases were collected 3 ± 2 months prior to the EDI, with individuals acting as controls matched 2:1 to cases based on sex, age, risk group, follow-up duration, and plasma sample availability.

#### Samples collected 6 ± 2 months prior to HIV infection.

Plasma samples collected 6 ± 2 months before the EDI were retrieved. Individuals acting as controls were matched 2:1 to HIV cases based on age, sex, risk group, study follow-up duration, and the availability of plasma samples collected within ±2 months of the index case’s calendar date.

### Isolation of exRNA

Nanofiltration and ultracentrifugation were used to isolate exRNA, primarily enriching for those encapsulated in small EVs, as described previously (19). In brief, 13.5 mL prefiltered PBS was combined with 300 μL plasma in a 15 mL Falcon tube. The diluted plasma was filtered through a 0.22 μm (Millipore) filter to exclude cell debris and centrifuged at 150,000*g* for 2 hours at 4°C without breaks. The pellets were treated with RNase A for 15 minutes and washed at 150,000*g* for 2 hours at 4°C. The impact of RNase treatment was evaluated by comparing the exRNA profile before and after treatment using a Bioanalyzer/Agilent TapeStation (Supplemental Figure 5). The supernatant was discarded, and the pellets were digested with 250 μL RNA lysis solution (Bioline) and stored at –80°C until needed. exRNA was extracted from lysed pellets using the Isolate II RNA Mini Kit (Bioline) according to the manufacturer’s instructions.

### Bead-assisted flow cytometry

Evaluation of small EV markers in our pellets was performed using bead-assisted flow cytometry (Supplemental Figure 6), as we previously described (19, 88). Briefly, 50 μL of EVs in PBS were incubated with 1 μL of aldehyde/sulfate latex beads (Invitrogen) in a total volume of 1 mL PBS for 12 hours at room temperature on a rotary mixer. Following incubation, 110 μL of 1 M glycine was added to block unreacted sites, and the mixture was incubated for an additional 30 minutes at room temperature. Beads were pelleted by centrifugation at 2,000*g* for 5 minutes and washed once with 1 mL PBS. The pellet was resuspended in PBS supplemented with 0.5% FBS (PBS + 0.5% FBS) and stained with 1× anti-CD9-APC (catalog 341648, BD Biosciences) and 1× anti-CD63-PE (catalog 55705, BD Biosciences). Negative controls included beads incubated with (a) antibody cocktail without EVs and (b) isotype control antibodies: PE mouse IgG1 (catalog 556650, BD Biosciences) and APC mouse IgG1 (catalog 550854, BD Biosciences). Stained beads were washed twice with 500 μL PBS + 0.5% FBS and pelleted by centrifugation at 2,000*g* for 10 minutes. Data acquisition was performed using a BD Fortessa flow cytometer.

### cDNA library preparation

We used our previous protocol (19, 88) to prepare the cDNA libraries for sequencing. Briefly, Superscript III (Invitrogen) was used to produce the first strand from the total exRNA. Before synthesizing the second strand, the first-strand reaction was cleaned using RNAcleanXP beads. dTTP was replaced with dUTP while synthesizing the second strand to generate double-stranded cDNA. The cDNA was fragmented, end-repaired, and ligated to adapters. The cDNA was treated with uracil-specific excision reagent (USER) enzyme (New England Biolabs), followed by 19 cycles of PCR amplification to add Illumina primers and increase yield. Sequencing was performed using the NextSeq 550 genome analyzer.

### Quantification of HPgV-1 using PCR

HPgV-1 RNA was converted to cDNA using Superscript III reverse transcriptase (New England Biolabs). HPgV-1–positive samples were detected by amplicon-targeted PCR amplification of the 5′ untranslated region with the antisense primer 5′-ATGCCACCCGCCCTCACCCGAA-3′ (nt 494–473, according to GenBank accession AY196904) and the sense primer 5′-AAAGGTGGTGGATGGGTGATG-3′ (nt 67–87) using Q5 High-Fidelity DNA Polymerase (New England Biolabs). Amplification conditions were 50°C for 59 minutes, 10 minutes at 94°C, then 35 cycles of 30 seconds at 94°C, 1 minute at 55°C, and 1 minute at 72°C, followed by 20 minutes at 72°C. First-round PCR products were used in nested PCR with the antisense primer 5′-CCCCACTGGTCYTTGYCAACTC-3′ (nt 362–341) and sense primer 5′-AATCCCGGTCAYAYTGGTAGCCACT-3′ (nt 107–131). After 35 cycles of 30 seconds at 94°C, 30 seconds at 55°C, and 1 minute at 72°C, PCR products were visualized by agarose gel electrophoresis for the presence of a 256 nt band.

### Statistics

Gene body read coverage depicted in Supplemental Figure 7 was calculated using the RSEQC tool (https://rseqc.sourceforge.net/). Transcript quantities, in units of raw read counts and transcripts per million, were estimated by aligning the data to the human transcriptome using salmon (https://combine-lab.github.io/salmon/) and tximport (https://bioconductor.org/packages/release/bioc/html/tximport.html). Comparison between cases and controls was performed using edgeR (https://bioconductor.org/packages/release/bioc/html/edgeR.html). Raw read counts were normalized using the relative log expression method, and the likelihood ratio test was chosen. *P* values were adjusted for multiple testing using the Benjamini-Hochberg procedure, and an FDR threshold of 5% was set as the cut-off for significance. Endotyping was performed using spectral clustering, and differences in gene expression between endotypes were determined using edgeR as described above. Cellular overrepresentation was performed using protein signatures derived from a previously published study (89), while pathway gene sets were obtained from Literature Lab (90) and Wikipathway (91). Pathogen classification was performed with Kraken2, and pathogen abundance comparisons between cases and controls were performed with edgeR. In parallel, the predictive value of HPgV-1, as measured by both sequencing and PCR, was also assessed by calculating risk ratios, with or without adjustment for other STIs. The HPgV-1 phylogenetic tree was generated by first performing a multiple sequence alignment with nextalign (https://github.com/neherlab/nextalign), followed by tree reconstruction with iqtree (https://iqtree.github.io/). Unless stated otherwise, all visualizations were carried out using ggplot2 (https://cran.r-project.org/web/packages/ggplot2/index.html) and ComplexHeatmap (https://bioconductor.org/packages/release/bioc/html/ComplexHeatmap.html) R packages. *P* values of less than 0.05 were considered significant.

### Study approval

The samples used in this study were collected using the IAVI protocol B, which was reviewed by the Kenya Medical Research Institute Ethical Review Committee, Nairobi, Kenya. Participants provided their written informed consent.

### Data availability

This study did not produce unique reagents or materials. RNA-seq data have been deposited at GEO under the accession number GSE287060 (https://www.ncbi.nlm.nih.gov/geo/query/acc.cgi?acc=GSE287060). Data point values for all graphs are available in the Supporting Data Values file.

## Author contributions

AIA, EWN, and TN jointly conceived the project and secured funding. PB guided data analysis and review of the manuscript. ES designed and ran the cohort and contributed to the study design and the manuscript review. LF and ASH participated in the study design and sample selection. MK and SM performed the laboratory experiments. MK performed data analysis and wrote the initial draft. JTB designed HPgV-1 primers and assisted in project conception. AIA and EWN jointly supervised the project. All authors read and reviewed the final draft.

## Funding support

- Wellcome Trust Award (209289/Z/17/Z).
- The Bill & Melinda Gates Foundation (INV-033558).
- Gilead Sciences Inc. (19275).
- Aidsfonds (0454).
- PEPFAR and USAID, through contributions by United States taxpayers, supported IAVI’s cohort study.
- SANTHE, as administered by the Africa Health Research Institute, through DELTAS Africa programme (Del-22-007), which is supported by the following.
- Science for Africa Foundation.
- Wellcome Trust.
- UK Foreign, Commonwealth & Development Office.
- European & Developing Countries Clinical Trials Partnership (EDCPT2) programme, which is supported by the European Union.
- Ragon Institute of Mass General Brigham, MIT, and Harvard.

## Supplementary Material

Supplemental data

ICMJE disclosure forms

Supplemental table 1

Supplemental table 2

Supplemental table 3

Supplemental table 4

Supplemental table 5

Supplemental table 6

Supporting data values

## Version 1 02/12/2026

In-Press Preview

## Version 2 04/01/2026

Electronic publication
