# Supplementary material for: Variation in antiviral immunity and inflammation pathways precedes HIV-1 infection in a high-risk African cohort
Source: J Clin Invest. 2026 Feb 12;136(7):e195172. doi: 10.1172/JCI195172 (PMC13038197; doi:10.1172/JCI195172)
Supplement: Supplemental data [file jci-136-195172-s137.pdf]

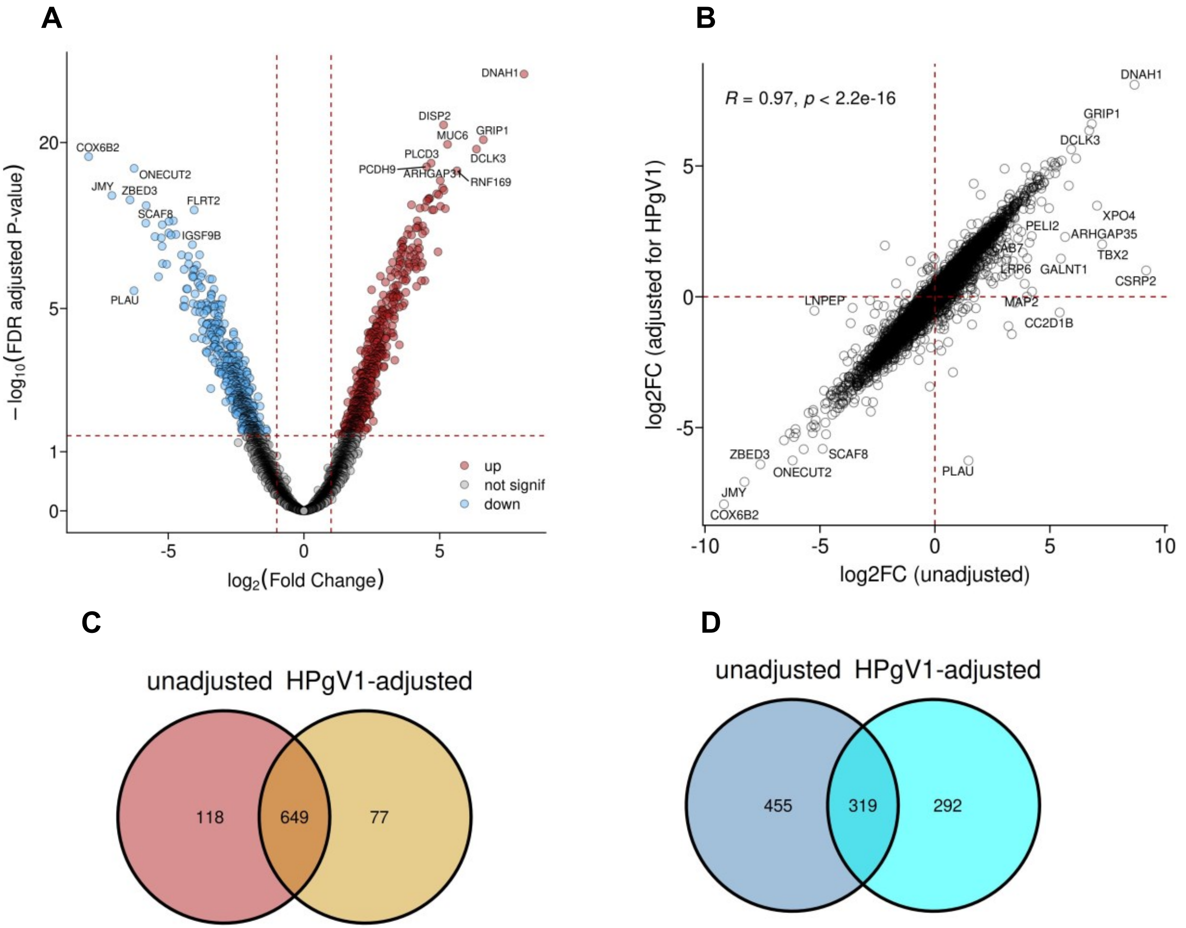

Figure S1: HPgV1 has minimal impact on transcriptome alterations between HIV1-cases and controls three months prior to infection. A) Volcano plot showing HPgV1-adjusted transcriptional alterations between cases and negative controls, three-months prior to HIV-1 infection. B) Scatterplot showing a high correlation between the log2FCs before and after adjusting for HPgV1. C) and D) Venndiagram showing overlap between upregulated (C) and downregulated (D) genes before and after adjusting for HPgV1.

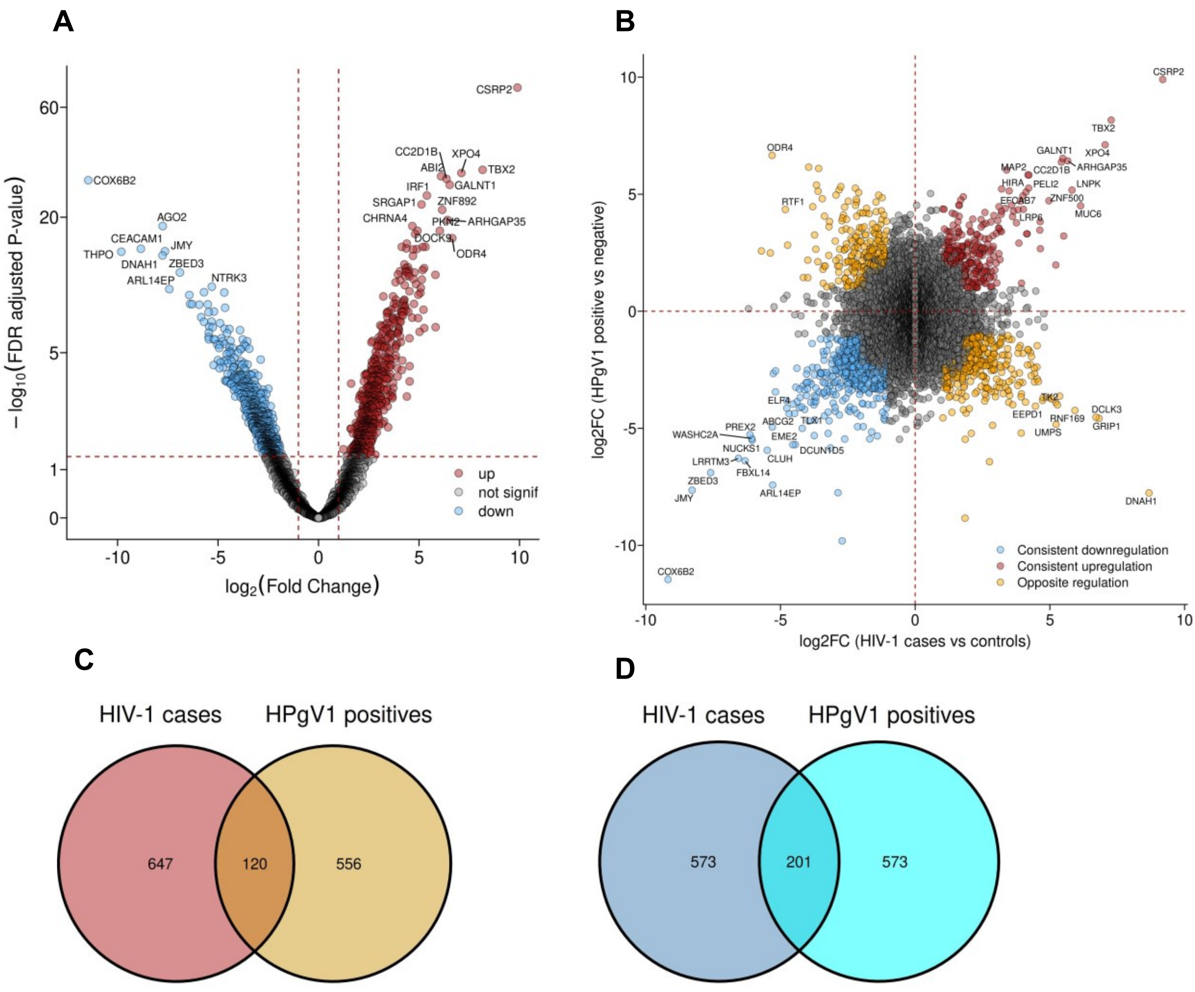

Figure S2: Modest consistency in differential expression changes between HIV-1 case-control comparison and HPgV1 positive-negative comparison. A) Volcano plot showing transcriptional alterations between HPgV1 positive and negative samples, three-months prior to HIV-1 infection. B) Scatterplot comparing the HIV1 case-control log2FC and HPgV1 positive-negative log2FC. C) and D) Venndiagram showing overlap between upregulated (C) and downregulated (D) in both comparisons.

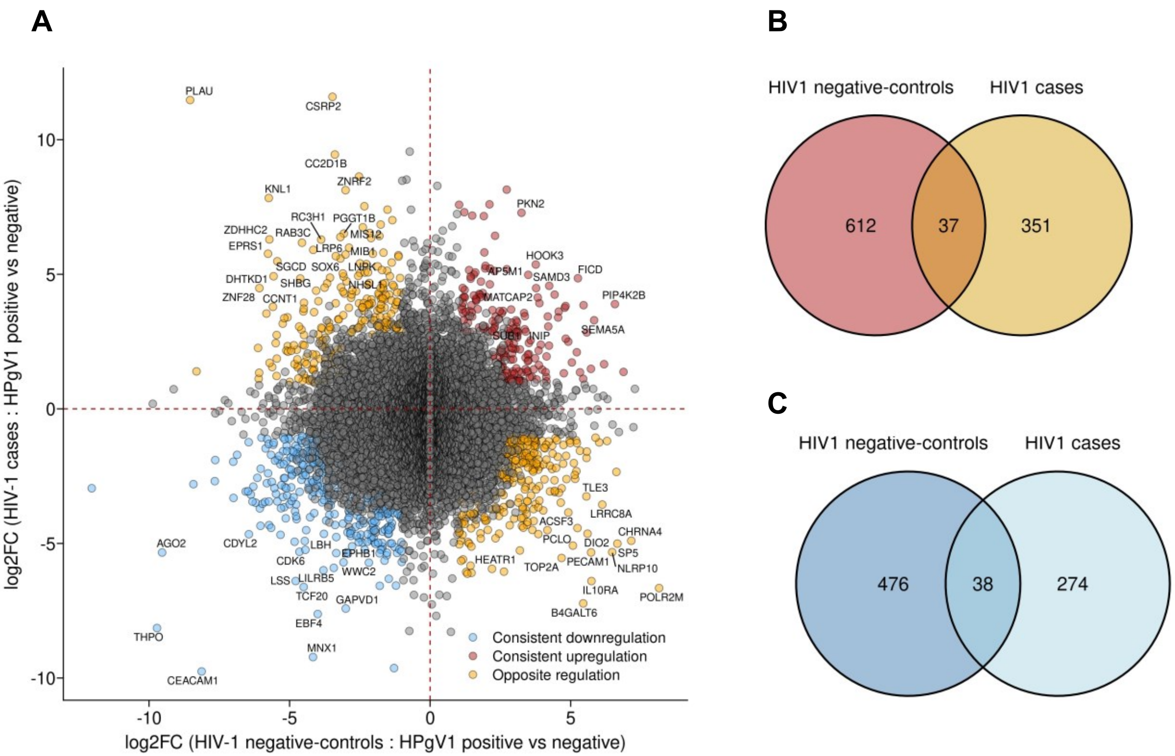

Figure S3: Little concordance between HPgV1 associated transcriptional patterns in HIV-1 cases and negative-controls. A) Scatterplot comparing HPgV1 associated gene expression alterations between HIV-cases and negative controls, . B) and C) Venndiagram showing overlap between upregulated (B) and downregulated (C) in both comparisons.

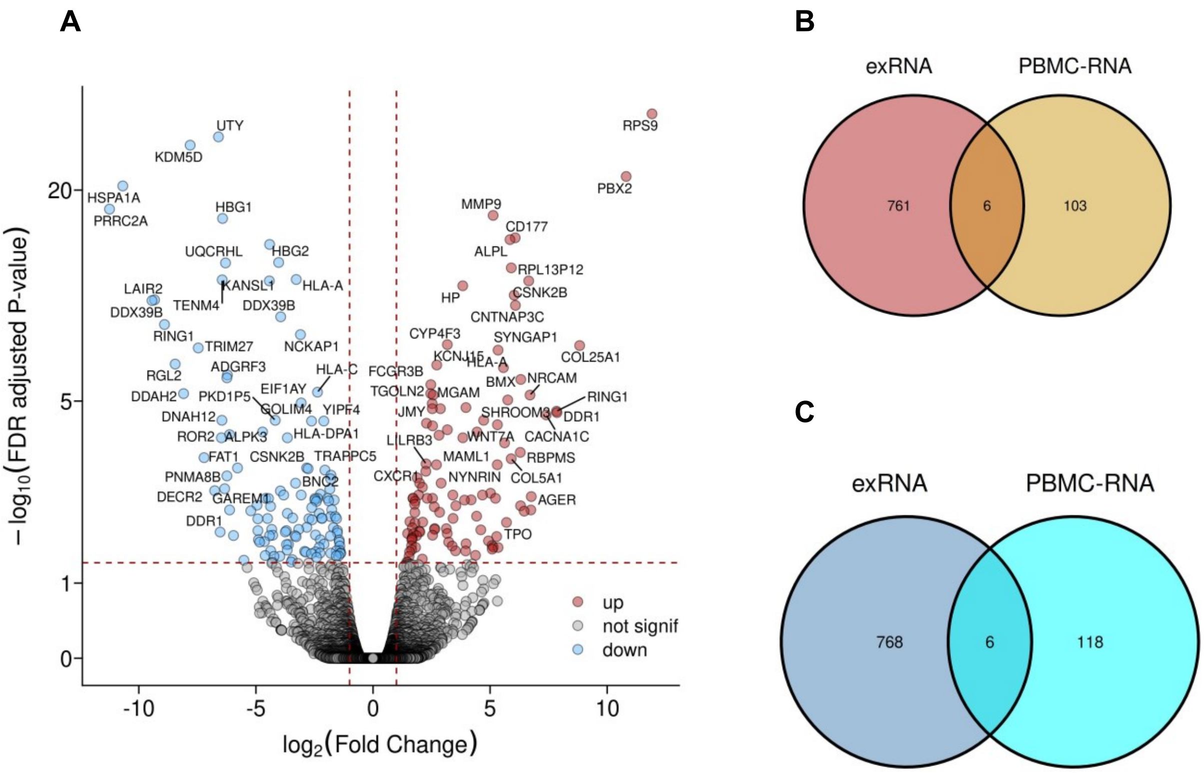

Figure S4: Little concordance between HPgV1 associated cell-free RNA patterns and HPgV1 associated PBMC RNA patterns. A) Volcano plot comparing HPgV1 associated gene expression alterations between PBMCs and cell-free transcriptomes. B) and C) Venndiagram showing overlap between upregulated (B) and downregulated (C) in both comparisons.

A1: S1_before_RNAse

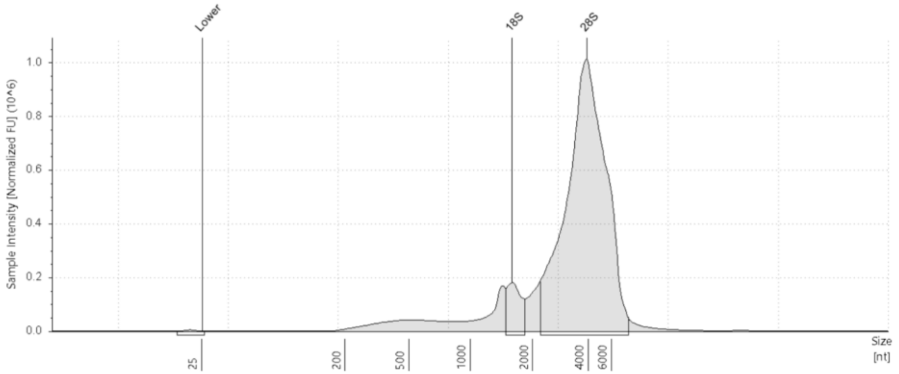

D1: S1_after_RNAse

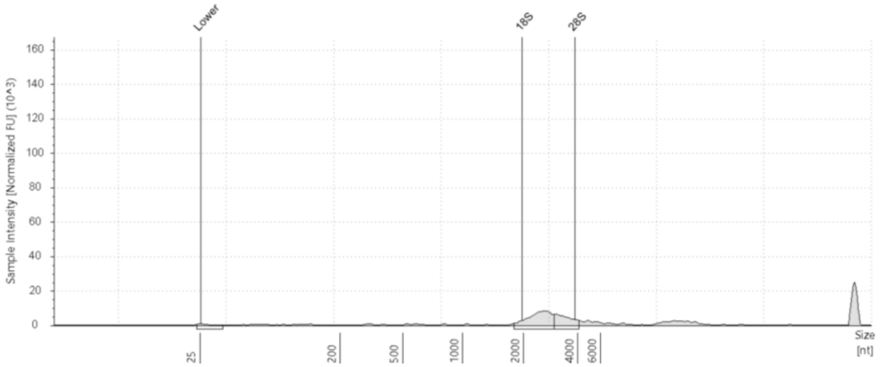

Figure S5: Degradation of RNA by RNAse A. We isolated cellular RNA and treated it with the same RNAse A we used to treat our pellets. The enzyme was working as expected.

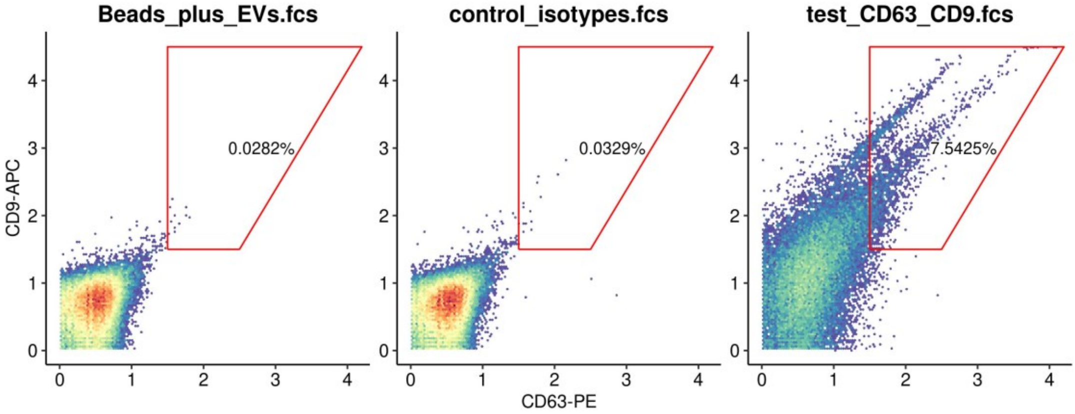

Figure S6: Plasma-derived ultracentrifugation pellets contain extracellular vesicles. We used bead-assisted flow cytometry to probe for EVs markers (CD63 and CD9) in the pellets obtained after high-speed centrifugation of processed plasma (150,000xg for 2 h). Left=beads were incubated with pellets alone without antibodies, middle=beads incubated with pellets followed by isotype control antibodies and right=beads incubated with pellets followed by anti-CD63 and anti-CD9.

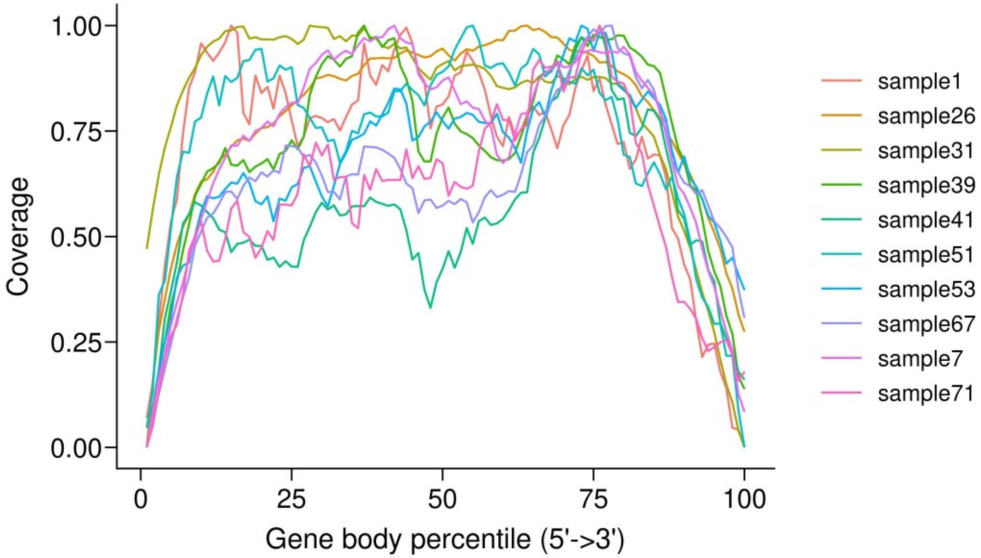

Figure S7: Read Coverage was globally uniform across the genes' length. We randomly selected 10 samples and calculated the gene body percentile coverage. There is no significant bias in read coverage.
